# Proteinase-Activated Receptor 2 May Drive Cancer Progression by Facilitating TGF-β Signaling

**DOI:** 10.3390/ijms18112494

**Published:** 2017-11-22

**Authors:** Hendrik Ungefroren, David Witte, Bernhard H. Rauch, Utz Settmacher, Hendrik Lehnert, Frank Gieseler, Roland Kaufmann

**Affiliations:** 1 First Department of Medicine, University Hospital Schleswig-Holstein, D-23538 Lübeck, Germany; davidwittemail@gmail.com (D.W.); Hendrik.Lehnert@uni-luebeck.de (H.L.); frank.gieseler@uksh.de (F.G.); 2 Department of General and Thoracic Surgery, University Hospital Schleswig-Holstein, D-24105 Kiel, Germany; 3 Department of General Pharmacology, Institute of Pharmacology, University Medicine Greifswald, D-17487 Greifswald, Germany; Bernhard.Rauch@uni-greifswald.de; 4 Department of General, Visceral and Vascular Surgery, Jena University Hospital, D-07747 Jena, Germany; Utz.Settmacher@med.uni-jena.de (U.S.); Roland.Kaufmann@med.uni-jena.de (R.K.)

**Keywords:** pancreatic carcinoma, signaling, TGF-β, ALK5, PAR2, serine proteinases

## Abstract

The G protein-coupled receptor proteinase-activated receptor 2 (PAR2) has been implicated in various aspects of cellular physiology including inflammation, obesity and cancer. In cancer, it usually acts as a driver of cancer progression in various tumor types by promoting invasion and metastasis in response to activation by serine proteinases. Recently, we discovered another mode through which PAR2 may enhance tumorigenesis: crosstalk with transforming growth factor-β (TGF-β) signaling to promote TGF-β1-induced cell migration/invasion and invasion-associated gene expression in ductal pancreatic adenocarcinoma (PDAC) cells. In this chapter, we review what is known about the cellular TGF-β responses and signaling pathways affected by PAR2 expression, the signaling activities of PAR2 required for promoting TGF-β signaling, and the potential molecular mechanism(s) that underlie(s) the TGF-β signaling–promoting effect. Since PAR2 is activated through various serine proteinases and biased agonists, it may couple TGF-β signaling to a diverse range of other physiological processes that may or may not predispose cells to cancer development such as local inflammation, systemic coagulation and pathogen infection.

## 1. Introduction

Late-stage pancreatic ductal adenocarcinoma (PDAC) and hepatocellular carcinoma (HCC) are non-curable diseases with a particularly poor prognosis. Over the last decade, research has increasingly focused on the microenvironment surrounding cancer cells, and its role in tumor growth and metastasis. Despite marked differences in their pathological features—PDAC are stromal-predominant, fibrotic/desmoplastic, poorly vascularized tumors, whereas HCC are cellular and highly vascularized—PDAC and HCC share the G protein-coupled receptor “proteinase-activated receptor 2” (PAR2) and transforming growth factor-β (TGF-β) as key factors involved in primary tumor growth and cancer cell invasion and metastasis. Recent results from our and other laboratories have shown a previously unexpected array of functional interactions between the PAR2 agonist/PAR2 and TGF-β/TGF-β receptor ligand-receptor systems that might be exploited by cancer cells to enhance tumorigenesis. In this review, we focus on one aspect of this signaling crosstalk, the impact of tumor cell and stromal cell-expressed PAR2 expression on TGF-β signaling and TGF-β-mediated cellular responses in PDAC-derived cancer cells and HCC-derived cancer and stromal cells.

## 2. PAR2 and TGF-β

### 2.1. PAR2 Signaling

**Figure 1:**
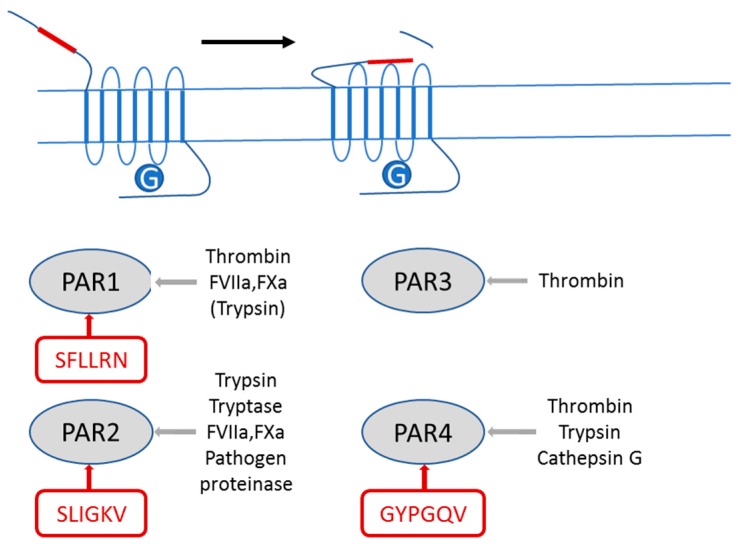
Diagram illustrating the mechanism of activation of proteinase-activated receptors (PARs) by proteinases and agonistic peptides. Each member of the family, PAR1, PAR2, PAR3 and PAR4, is activated by the indicated serine proteinases (in grey). Alternatively, each PAR may be activated in a cleavage-independent manner by peptide sequences (boxed in red) from its tethered ligand region (red lines in the cartoon at the top).

PAR2 is a prototype member of a subfamily of G protein-coupled receptors, the proteinase-activated receptors (PARs) that also comprise PAR1, PAR3 and PAR4. These receptors regulate a variety of physiological processes such as vasoregulation, nociception, inflammation [1,2,3,4] as well as a number of pathologies including tissue fibrosis [5] and cancer [6,7,8,9,10,11,12,13]. PARs are activated by serine proteinase-mediated cleavage of the extracellular N-terminus resulting in exposure of a “tethered ligand” that binds to the receptor and activates it [1,2] (Figure 1). Each of the four PARs is activated by a specific though overlapping set of serine proteinases (Figure 1). The principal enzyme activator for PAR2 is trypsin, which cleaves PAR2 at its “canonical” R//S tethered ligand-generating activation site. Besides trypsin, PAR2 is activated by tissue factor (TF)/factor VII/VIIa (FVII/VIIa) /factor Xa (FXa) complexes. TF, a transmembrane glycoprotein bound to coagulation serine proteinase factor VII/VIIa (FVII/VIIa), is a major activator of the coagulation system in cancer patients. The TF/FVIIa complex is formed on the surface of extracellular vesicles released by tumor cells and immune cells in an inflammatory microenvironment. It significantly aggravates the procoagulatory situation with deleterious consequences for cancer patients and can induce tumor cell migration through PAR2 signaling [14]. In vitro PARs can also be activated with short synthetic peptides derived from the proteolytically-exposed “tethered ligand”, so-called PAR-activating peptides (PAR-APs) that are capable of PAR-specific activation without receptor proteolysis (Figure 1). These PAR-APs have proved to be useful tools to study the function of PARs, particularly in settings in which more than one PAR subtype is expressed and stimulated by the same proteinase.

Following activation, PAR2 engages G_αq_, G_αi_ and G_α12/13_ subtypes and triggers mobilization of Ca^2+^ from intracellular stores and activation of extracellular-regulated kinase (ERK)1/2, RhoGEF-mediated Rho and Rac signals [1], but it can also signal independently of G proteins via the multifunctional adapter protein β-arrestin-2 [1,3]. As a third possibility, PAR2 may homo- and heterodimerize, transactivate receptor tyrosine kinases (RTKs) and receptor serine/threonine kinases (RSTKs), or communicate with other G protein-coupled receptors, toll-like receptors, nucleotide-binding oligomerization domain (NOD)-like receptors, or ion channel receptors (for a comprehensive review of these functional receptor interactions and their physiological and pathological roles, see [2]).

### 2.2. The Role of PAR2 in Pancreatic and Liver Cancer

In the liver and pancreas PAR2 is involved in fibrosis [5,15] and the desmoplastic reaction seen in pancreatic cancer tissues. The tumor microenvironment is known to be crucial for tumor growth and metastasis and consists of the tumor cell and the stromal compartment. The latter is composed of a variety of stromal cells such as myofibroblasts/stellate cells, cancer-associated fibroblasts and various types of immune cells [16,17]. Interactions of tumor cells with stromal cells are thought to promote primary tumor growth and metastatic spread. They involve both cell-to cell interactions and communication through PAR2-activating serine proteinases—secreted by both cancer and stromal cells—and a plethora of soluble factors including the TGF-βs [16,17].

Although in skin tumorigenesis PAR2 has been suggested to be a tumor suppressor [18], this member of the PAR family has mostly been associated with cancer development and progression because of its ability to control tumor growth, invasion and metastasis in various tumor entities including hepatocellular carcinoma (HCC) and PDAC [6,7,8,9,10,11,12,13]. Pancreatic cancer cells express high levels of PAR2 and activation of PAR2 induces their proliferation, migration and invasion [9,10,13,15]. However, in colorectal cancer, PAR2 (and PAR1) were both expressed in normal stroma but demonstrated increased expression in cancer stroma. This may point to an important role of these PARs in the stromal compartment, which is further confirmed by the finding that the respective agonists—TF and thrombin—were both expressed by cancer but not normal stroma [18]. PAR2 expression was also found to be increased in stroma-rich pancreatic cancer regions, together suggesting a potential role of PAR2 in the tumor microenvironment. Functional proof for that came from a study in an orthotopic pancreatic cancer model, in which tumor cells are PAR2-positive, whereas stromal cells are PAR2-negative [13]. Genetic ablation of *F2RL1* from the stromal compartment inhibited primary tumor growth and was accompanied by reduced vascularization in primary tumors and a reduction in tube formation of vascular endothelial cells in vitro. The smaller primary tumors contrasted with an increased number of lymphatic vessels and lymph node metastases in the PAR2-deficient animals. In vitro tube-formation assays showed that PAR2 inhibited cancer cell-induced tube formation. Stromal PAR2 therefore has a dual function in pancreatic cancer development, potentiating primary tumor growth but limiting lymphangiogenesis and subsequent lymph node metastasis. In another study with *F2RL1*^−/−^ mice and subcutaneously inoculated B16 melanoma cells, the opposite effect was observed: growth of larger primary tumors but generation of less distant metastases, which allowed for a longer survival time of their hosts compared with strain-matched wild-type controls [12]. These data suggest that host stromal cell-derived PAR2 inhibited primary tumor growth but enhanced metastatic spread. The reason for this discrepancy may the mode of application (orthotopic vs. subcutaneous) or the different tumor types (pancreatic cancer vs. melanoma). However, in gastric cancer, PAR2 expression correlates with the depth of wall, lymphatic and venous invasion as well as with liver metastasis. In agreement with this, patients with PAR2-positive tumors had a significantly poorer prognosis than patients with PAR2-negative tumors [11].

Our group analyzed the role of stromal cell-derived PAR2 from hepatic stellate cells (HSCs) for HCC growth [19]. In vivo studies showed that the HSC cell line LX-2 promoted tumor growth and angiogenesis of HCC xenografts in mice. These effects were significantly reduced when PAR2 was downregulated by RNAi. In vitro studies demonstrated that inhibition of PAR2 attenuated SMAD2/3 activation in response to TGF-β1 stimulation in LX-2 cells and blocked the pro-mitotic effect of LX-2-derived conditioned medium on Hep3B cells. Moreover, PAR2 stimulation of LX-2 cells with trypsin or a PAR2-AP resulted in the activation of different signaling pathways, increased secretion of pro-angiogenic and pro-mitotic factors and proteinases, and enhanced migration. RNAi-mediated depletion of PAR2 or pharmacological inhibition of Src, MET, platelet-derived growth factor receptor (PDGFR), ERK1/2 mitogen-activated protein kinase (MAPK), or matrix-metalloproteinases (MMPs) blocked PAR2-AP-induced migration. HSC-derived PAR2 thus plays a crucial role in promoting HCC growth and its targeting may have therapeutic relevance in HCC [19].

Ductal carcinoma in situ (DCIS) is a pre-invasive breast cancer and genotypic changes have been identified in stroma surrounding DCIS. Stromal fibroblasts undergo a phenotypic change in cancer to promote tumor angiogenesis, proliferation, immunosuppression and metastasis and in vivo can induce invasion of DCIS. Phenotypic changes in DCIS stromal fibroblasts may potentially act as a precursor for invasion. Interestingly, procoagulant phenotypic changes with increased tissue factor, thrombin and PAR2 expression have been observed in stromal fibroblasts at the pre-invasive stage; however, it has yet to be determined if this change is functional and whether it has potential as a therapeutic target for preventing transition to invasion [20].

### 2.3. TGF-β Signaling

TGF-β is a member of the superfamily of the TGF-β/activin/bone morphogenetic protein (BMP) family of ligands. There are three isoforms (TGF-β1-3), all of which signal through two highly glycosylated, membrane-bound receptors termed type II (TβRII) and the type I receptor “activin receptor-like kinase 5” (ALK5). Following ligand binding and phosphorylation by TβRII on Ser/Thr residues, ALK5 triggers activation of canonical SMAD signaling. ALK5-mediated phosphorylation/activation of SMAD2 and SMAD3 at their C-terminus (SMAD2C/SMAD3C) is a crucial step in the control of TGF-β signaling pathway activity. Activated SMAD2/3 subsequently bind to the common mediator SMAD, SMAD4, and the complexes of SMAD2/SMAD4 and SMAD3/SMAD4 are translocated to the nucleus, where they bind to the promoters of TGF-β target genes to regulate their transcription [21,22].

TGF-β also eventually stimulates activation of SMAD-independent pathways to regulate cell motility, e.g., MKK3/6-p38 and MEK-extracellular signal-regulated kinase 1/2 (ERK1/2) mitogen-activated protein kinase (MAPK) signaling [23]. Depending on the cell type, activation of these non-canonical pathways can be SMAD-dependent or -independent. Activation of ERK by TGF-β1 is quite variable among cell types and is often delayed and moderate. It can originate directly through the tyrosine kinase function of ALK5 [24] or via Ras and Raf through epidermal growth factor receptor (EGFR) transactivation [25]. For instance, in mesangial cells, TGF-β induces EGFR autophosphorylation resulting from HB-EGF release and binding, and this ligand release-dependent EGFR transactivation is rapid and transient in these cells [26]. Another example of TGF-β1-EGFR crosstalk was provided for fibroblast to myofibroblast differentiation, where hyaluronan (HA) facilitates TGF-β1-dependent fibroblast differentiation through HA-CD44 binding and relocation, promoting interaction between CD44 and EGFR within lipid rafts [27]. Crosstalk between PDGF/PDGFR autocrine signaling loop with TGF-β/ALK5 has been described to promote metastatic dissemination of mammary tumors [28]. There is evidence that TGF-β1-induced angiogenesis acts with vascular endothelial growth factor (VEGF) to mediate apoptosis of excessive vascular sprouts and may even be required for initial sprouting from an existing vascular network [29]. In addition, TGF-β1 induces a dose-dependent inhibition of endothelial cord formation and modulation of endothelial VEGFR2 expression in an ALK5-dependent fashion [30]. Finally, several studies reveal that hepatocyte growth factor (HGF)/MET signaling axis antagonizes the profibrotic actions of TGF-β1 by intercepting SMAD signal transduction through diverse mechanisms [31,32,33]. In addition, TGF-β1/ALK5 and HGF/Met negatively regulate each other’s production during kidney injury [34]. If one considers that numerous studies provide evidence for PAR2 transactivation of EGFR, VEGFR, PDGFR and MET (for review see [2]), it seems quite likely that TGF-β1/ALK5 and PAR2 can cooperatively signal via these RTKs.

### 2.4. The Role of TGF-β in Pancreatic and Liver Cancer

Besides breast carcinoma, PDAC and HCC represent tumor types in which the crucial role of TGF-β as a tumor promoter is well documented [35]. In PDAC the aggressive nature is caused in part by somatic mutations in *KRAS*, *p16^INK4A^*, *TP53* and *DPC4*. *DPC4*, encoding the SMAD4 protein, is a central mediator of TGF-β signaling and is mutated in ~50% of invasive pancreatic carcinomas [36]. TGF-β signaling has a central role in the cancer progression of PDAC because (i) the TGF-β pathway naturally contains mutations and other well-defined alterations, and (ii) it belongs to only four signaling pathways that are mutated in 100% of tumors [37]. In addition, mouse models of PDAC have shown that mutations in the TGF-β pathway, e.g., in SMAD4 and TGF-β type II receptor, are causative in the development of aggressive PDAC by cooperating with members of the K-Ras and other pathways to induce neoangiogenesis, host immune suppression, invasion and metastasis [38]. Like PAR2, TGF-β has been associated negatively with tumor growth and positively with invasion and metastasis. With respect to primary tumor growth orthotopic xenografts of the human PDAC cell line Panc1 expressing a kinase-active mutant of ALK5 in *scid*/*bg* mice presented with smaller primary tumors while generating more distant metastases [39]. PDAC-derived cells display a particularly high expression of both PAR2 and TGF-β1 and in advanced cancer stages TGF-β can auto-induce its expression. This eventually results in accumulation of TGF-β in the tumor tissue through a positive feedback loop and eventually escalation of angiogenesis, EMT, migration, invasion and metastasis formation [40].

In addition to proinflammatory cytokines, TGF-β is considered to be a major factor promoting liver carcinogenesis [35,41]. It is overexpressed in HCC [42,43] and may also drive transformation of hepatic stellate cells (HSCs) into myofibroblasts [44,45], as well as their migration and invasion [46]. Since the human HSC line LX-2 is sensitive to TGF-β treatment [47] and PAR2 has been shown to stimulate TGF-β gene expression and protein production in HSCs [5], a PAR2–TGF-β interaction may also be suggested in HSC/LX-2 cells. Thus, PAR2 deficiency in LX-2 cells may also severely compromise TGF-β sensitivity of these cells towards stimulation with both autocrine and paracrine TGF-β.

## 3. Evidence for PAR and TGF-β/ALK5 Signaling Crosstalk

PAR2 and TGF-β signaling have overlapping functions such as the profibrotic role in the liver [5], the ability to stimulate profibrogenic cytokines, to induce the proliferation and differentiation of fibroblasts into myofibroblasts and to stimulate production of matrix proteins in HSCs in vitro and in vivo [5]. In addition, some intracellular signaling pathways and signaling intermediates are activated by both PAR2 and TGF-β/ALK5, e.g., ERK1/2 MAPK, p38 MAPK, PKC, Rac/Rho, c-Src, nuclear factor κB, reactive oxygen species and intracellular Ca^2+^ [1]. Moreover, PAR2 and TGF-β1 mutually regulate their expression with PAR2 inducing the synthesis of TGF-β1 in HSCs [5] and TGF-β1 upregulating PAR2 expression in endometrial stromal cells [48]. TGF-β1 and various PAR2-activating serine proteinases are rich constituents of the tumor microenvironment, where they mediate the dialogue between cancer cells and neighboring stromal cells [16,17]. The colocalization, their mutual regulation, the similar (patho)physiological functions and signaling properties suggested the possibility of a close functional interaction between TGF-β1/ALK5 and PAR2 signaling. The first direct evidence for this came from a study showing that peptide agonist-mediated activation of PAR2 and expression of connective tissue growth factor (CTGF) in human kidney epithelial cells was dependent on ALK5 and involved the activation of SMAD2 [49].

## 4. PAR2 and Its Requirement for TGF-β1-Mediated Cellular Responses and TGF-β/ALK5-Induced Signaling

### 4.1. TGF-β/ALK5-Induced Signaling

Many TGF-β-dependent responses are controlled at the transcriptional level; this is reflected in alterations in mRNA levels of TGF-β-target genes. Promoter reporter gene assays in Panc1 and HEK293T cells with TGF-β-responsive plasmids that are entirely (p6SBE-Luc, p(CAGA)_12_-Luc) or partially (p3TP-Lux) SMAD-dependent showed that after siRNA-mediated silencing of PAR2, the TGF-β1-mediated transcriptional activity of all three reporters was either lost or severely reduced. This suggests that PAR2 is required for the general SMAD-based transcriptional activation by TGF-β1 [50]. Given the decrease in SMAD-mediated transcription upon PAR2 depletion, we sought to reveal whether PAR2 affects canonical SMAD signaling, specifically activation of receptor-regulated SMADs, SMAD2 and SMAD3. Interestingly, PAR2-depleted Panc1, Colo357, and IMIM-PC1 cells were no longer able to respond to TGF-β1 with C-terminal phosphorylation of SMAD3 and SMAD2 [50]. Together, these findings strongly suggest that a functional cooperation between the TGF-β receptor(s) and PAR2 is required for SMAD activation and signaling.

TGF-β signals through the canonical SMAD pathway as well as through non-canonical pathways such as p38 and ERK MAPKs, phosphotidyinositol-3-kinase/AKT, and Rho/Rac-dependent pathways. While SMAD signaling mainly accounts for the tumor-suppressive functions of TGF-β, the non-SMAD pathways predominate during later stages of tumor development when the SMAD pathway is often inactivated, e.g., by mutations in *DPC4*, to mediate the tumor-promoting functions of TGF-β. It was therefore of interest to study the involvement of PAR2 in MKK3/6-p38 and MEK-ERK1/2 signaling, both of which cooperate with SMAD signaling in TGF-β1-induced cell motility. Intriguingly, PAR2-depleted cells failed to activate p38 MAPK in response to TGF-β1 stimulation, as evidenced by defective phosphorylation on Thr180/Tyr182 [50]. Moreover, TGF-β target genes that rely on p38 signaling for full induction by TGF-β1 such as biglycan [51] were refractory to TGF-β1 stimulation in cells depleted of PAR2 protein. With respect to MEK-ERK signaling, we recently observed in HaCaT cells (which activate ERK1/2 at 1–2 h after application of TGF-β1) a severe decrease in phospho-ERK1/2 levels at the 1 h time point following siRNA-mediated silencing of *F2RL1* [52].

### 4.2. Cell Migration and Invasion

Based on the observation that PAR2 agonistic peptide-mediated induction of CTGF expression in human kidney cells was dependent on ALK5 [49], we pursued the possibility that signaling by TGF-β1 is, in turn, dependent on PAR2. Using siRNA-mediated silencing of PAR2 and real-time analysis of random cell migration and invasion, we have verified that PAR2 protein expression was required for TGF-β1-mediated cell motility in several (TGF-β-sensitive) cell lines of PDAC origin [50]. In search of the molecular basis, we found that PAR2 depletion blunted the TGF-β response of several TGF-β target genes involved in the regulation of cell motility, including the prototypical TGF-β response gene *serpine1*, encoding plasminogen activator inhibitor-1 (PAI-1) [50].

### 4.3. Epithelial–Mesenchymal Transition (EMT) and EMT-Associated Alterations Relevant for Therapy

EMT is considered a prerequisite for cells to become invasive and eventually metastatic. Since PAR2 expression is crucial for TGF-β1-dependent migration and invasion, PAR2 may also be involved in TGF-β1-induced EMT and EMT-associated changes such as proliferation arrest, acquisition of a chemoresistant phenotype and possibly even the generation of cancer stem cells (CSC). In fact, we found that cellular depletion of PAR2 from PDAC cells and HaCaT keratinocytes by RNAi resulted in a greatly decreased response of several EMT and stem cell marker genes to TGF-β1 stimulation [53]. Moreover, cells depleted of PAR2 protein lost the ability to auto-induce endogenous TGF-β1 in response to stimulation with exogenous TGF-β1. This suggests that inhibiting PAR2 expression or function in vivo is a promising strategy to disrupt a TGF-β auto-stimulatory loop in the tumor tissue that is believed to escalate the malignant phenotype [40]. A decrease in proliferation is part of the EMT program. Intriguingly, we noted that growth inhibition by TGF-β1 was lost in PAR2-depleted Panc1 and Colo357 cells [53]. The failure of TGF-β1 to induce growth arrest in PAR2-deficient cells was associated with its inability to induce the cyclin-dependent kinase inhibitor p21^CIP1/WAF1^ [53].

## 5. PAR2 Signaling Functions Required for Promoting TGF-β Signaling

As mentioned above, PAR2 binds to G_αq_, G_αi_ and G_α12/13_ subtypes and triggers mobilization of intracellular Ca^2+^ and various other signaling pathways. To reveal whether G_q_-Ca^2+^ signaling is required for PAR2 to facilitate TGF-β signaling, we examined the effects of PAR2-activating peptides, PAR2 mutants and PAR2 inhibitors on various TGF-β1-induced responses. Stimulation of cells with PAR2 activating peptide alone was unable to enhance basal or TGF-β1-induced SMAD3C phosphorylation, SMAD-dependent transcriptional activity, and chemokinesis [54]. Likewise, in complementary experiments, abrogation of PAR2-G_q_-calcium signaling failed to inhibit TGF-β1-induced migration, reporter gene activity, and SMAD3 activation [54]. Our results so far suggest that although reducing PAR2 protein expression may potentially block TGF-β’s prooncogenic function, particularly migration/invasion [50], inhibiting its G_q_-calcium signaling arm alone would not be sufficient to achieve this effect.

## 6. Molecular Mechanism of the PAR2–TGF-β Interaction

**Figure 2:**
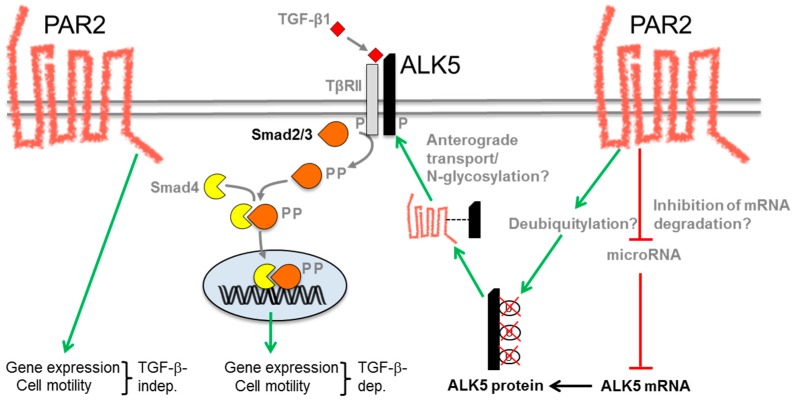
Schematic diagram illustrating the potential mechanisms of the PAR2–TGF-β/ALK5 interaction. On the left-hand side of the figure, the direct, TGF-β-independent (TGF-β-independent) effects of PAR2 on gene expression and cell motility are shown. On the right-hand side, hypothetical mechanisms of PAR2 function are summarized that may result in an increase in ALK5 abundance and/or membrane localization such as inhibition of an ALK5-targeting microRNA, ALK5 deubiquitylation or anterograde transport (plus *N*-glycosylation) of ALK5 from the Golgi apparatus to the cell surface. An increase in ALK5 expression eventually results in an enhancement of TGF-β-induced Smad2/3C phosphorylation and TGF-β/Smad-dependent (TGF-β-dep.) gene expression and cell motility. Stimulatory interactions are indicated by green arrows and inhibitory interactions by red lines. The physical interaction between intracellular PAR2 and ALK5 is marked by a black stippled line. P, phosphate residue.

Since SMAD2/3, p38 MAPK and ERK1/2 MAPK are all activated in a TGF-β receptor-dependent manner, we hypothesized that PAR2 could affect the expression or activity of either the ALK5 or the TβRII kinase. Upon monitoring the abundance of both receptors at the protein and mRNA level, we realized a profoundly lower expression of ALK5, but not TβRII, in cells in which *F2RL1* was silenced by RNAi, suggesting that PAR2 specifically targets ALK5. Rescue experiments with ectopic expression of either wild-type ALK5 or a kinase-active mutant (ALK5^T204D^) but not a SMAD binding-defective kinase-active mutant (RImL45^T204D^) was able to restore the cells’ sensitivity to TGF-β as seen by their ability to phosphorylate SMAD3C, activate a Smad-responsive reporter gene and resume cell migration in response to TGF-β1 stimulation [50]. We concluded that PAR2 facilitates TGF-β signaling by maintaining expression of ALK5. Currently, we are trying to unravel whether this occurs at the mRNA level through an increase in de novo transcription of *TGFBRI* or, alternatively, via suppression of a microRNA (miRNA) that targets ALK5 mRNA for degradation. Preliminary results from reporter gene assays using a *TGFBRI* promoter–luciferase construct (-392-pGL4) failed to yield evidence for de novo transcription as the underlying mechanism of PAR2-induced enhancement of ALK5 expression [53]. Rather, we favor another mechanism, namely regulation by a miRNA through the 3′-UTR of the ALK5 mRNA. To date, approx. 114 miRNAs have been described that control ALK5 expression. A literature search yielded two miRNAs that have been implicated in both ALK5 and PAR2 function (let7 and miR-200). We are currently analyzing regulation of both miRNAs by PAR2 in Panc1 and Colo357 cells by qPCR and their effect on ALK5 expression by miRNA antagomirs. More recently, we asked whether a physical interaction between PAR2 and ALK5 may exist. Data from co-immunoprecipitation experiments with ectopic expression of tagged versions of PAR2 and ALK5 have shown that both receptors interact physically although it remains to be seen whether this interaction is direct or whether they are part of a larger complex and what the functional significance is. PAR2 has been shown to act together with PAR1 as a functional unit during malignant and physiological invasion [55]. Another study has identified a novel regulatory role for PAR2 in the anterograde traffic of PAR4 [56]. Specifically, co-expression of PAR4 with PAR2 induced heterodimerization between both PARs and facilitated plasma membrane delivery of PAR4, an effect produced through disruption of β-COP1 binding and facilitation of interaction with the chaperone protein 14-3-3ζ. PAR2 also enhanced glycosylation of PAR4 and activation of PAR4 signaling [56]. We envisage a scenario in which PAR2 facilitated anterograde transport of ALK5. As for PAR4, PAR2 may also promote glycosylation of ALK5. In favor of this model are the data from co-immunoprecipitation experiments and the observation that G_q_-Ca^2+^ signaling (and other signaling functions) of PAR2 are dispensable for the TGF-β cooperative effect. The current ideas on the mechanistic interaction between PAR2 and TGF-β signaling are depicted in Figure 2.

## 7. Potential Implications of PAR2 Inhibition in Anti-Cancer Therapy

Based on our findings regarding the crucial role of PAR2 in TGF-β signaling, we would like to speculate on how disruption of the PAR2–TGF-β interaction could translate into future therapeutic strategies for PDAC and HCC. In light of the potent pro-oncogenic effects of TGF-β and their dependence on PAR2, inhibiting PAR2 expression or function in either the tumor or the stromal compartment of PDAC and HCC tumors is likely to have a strong albeit differential impact on tumor growth and metastasis: blocking PAR2 in the tumor cells or the stromal cells will slow down primary tumor growth *and* metastasis, blocking only stromal PAR2 in pancreatic cancer and HCC, although interfering with primary tumor growth in the case of PDAC, will be at the expense of enhanced lymphangiogenesis and possibly lymph node metastasis [13].

Inhibition of PAR2 may be a very effective approach involving on the one hand inhibition of PAR2-driven (and TGF-β-independent) invasion in response to activation by serine proteinases including blood coagulation enzymes like TF-FVII-FXa [57] and on the other hand disruption of prometastatic ALK5-dependent signaling in response to the high levels of TGF-β present in the tumor tissue. Moreover, both PAR2 [58] and TGF-β/ALK5 [59] promote angiogenesis through expression and release of VEGF and subsequent VEGFR transactivation and are thus essential for tumor survival under hypoxic conditions. In addition, PAR2 maintains a constitutive high level of HIF-1α to promote angiogenesis, which might explain the high propensity for metastatic spread of pancreatic cancer cells to regions of hypoxia [59]. We have also shown that, following PAR2 depletion, TGF-β1 was unable to upregulate several matrix-encoding genes in vitro [50], suggesting the possibility that inhibitors of PAR2 can interfere with the profibrotic effects of TGF-β in vivo and hence the desmoplastic reaction.

Several small-molecule PAR2 antagonists are now available that were based on the tethered ligand motif of PAR2 and that block receptor activation by trypsin (reviewed in [60]). These include two peptides (pepducins) and the compounds ENMD-1068, K-14585 and GB88. ENMD-1068 attenuates PAR2-mediated murine joint inflammation in vivo, while K-14585 and GB88 inhibit PAR2-dependent Ca^2+^ and pro-inflammatory signaling with attenuation of inflammation in a rat model of colitis. K-14585 and GB88 are “biased antagonists” as they inhibit PAR2-induced release of intracellular Ca^2+^, cyclic AMP stimulation, receptor internalization and pro-inflammatory cytokine release but not PAR2-mediated ERK MAPK phosphorylation [60]. As outlined above, we have found in pancreatic cancer cells that GB88 can even enhance TGF-β1-induced ERK activation [52] and cell migration [52,54], which is in line with the crucial role of MEK-ERK signaling in TGF-β-dependent invasion in these cells [61]. Hence, applying GB88 or PAR2 modulators derived from GB88 [62] to cancer patients runs the risk of exacerbating the metastasis of tumor cells.

Recently, an antagonist, AZ8838, has been developed that binds in a fully occluded pocket near the extracellular surface and exhibits slow binding kinetics, which is an attractive feature for a PAR2 antagonist competing against a tethered ligand. Another antagonist, AZ3451, binds to a remote allosteric site outside the helical bundle [63].

These newly developed PAR2 antagonists may be applied alone or in combination with TGF-β signaling inhibitors that are already in clinical use for anti-cancer therapy of advanced PDAC and HCC tumors [64]. A combination of both drugs is likely to be more effective in suppressing tumor progression by halting primary tumor growth through their effects on neoangiogenesis and fibrosis/desmoplasia. However, it might be necessary to target specifically tumor cell-derived PAR2 as stromal PAR2 has been shown to be beneficial in that it can limit lymphangiogenesis and subsequent lymph node metastasis [13]. Another strategy would be the disruption of the mutual regulation and auto-induction loops of TGF-β and PAR2 expression [5,48]. Moreover, it has been shown that PAR2 agonist-dependent CTGF expression in renal cells is ALK5-dependent [49]. If PAR2 transactivation of ALK5 also contributes to PAR2 agonist-dependent invasion and metastasis, then small molecule inhibitors of the ALK5 kinase, such as SB431542, LY2157299 or SD-208 [64], would further potentiate a combined TGF-β + PAR2 inhibition strategy.
